# Occurrence of Strongylid Nematode Parasites on Horse Farms in Berlin and Brandenburg, Germany, With High Seroprevalence of *Strongylus vulgaris* Infection

**DOI:** 10.3389/fvets.2022.892920

**Published:** 2022-06-10

**Authors:** Laura Jürgenschellert, Jürgen Krücken, Eric Bousquet, Jürgen Bartz, Nina Heyer, Martin K. Nielsen, Georg von Samson-Himmelstjerna

**Affiliations:** ^1^Institute for Parasitology and Tropical Veterinary Medicine, Freie Universität Berlin, Berlin, Germany; ^2^Virbac, Carros, France; ^3^Virbac Tierazneimittel GmbH, Bad Oldesloe, Germany; ^4^M.H. Gluck Equine Research Center, University of Kentucky, Lexington, KY, United States

**Keywords:** nematodes, equine parasites, large strongyles, ELISA, *Strongylus* spp.

## Abstract

The infection of horses with strongylid nematodes is highly prevalent, with multi-species infections being the rule. *Strongylus* spp. and in particular *Strongylus vulgaris* are amongst the most pathogenic strongyle equine parasites. Presumably due to regular strategic anthelmintic treatments in combination with long prepatencies, prevalence of these worms was severely reduced in past decades. In this study, 484 horses from 48 farms in Berlin/Brandenburg, Germany were sampled between May 2017 and January 2018. Mini-FLOTAC and combined sedimentation/flotation were used to analyse faecal samples and larval cultures were carried out from individual strongyle infected horses for molecular testing for *Strongylus* spp. infection. Additionally, for *Strongylus vulgaris*, antibodies against a recombinant larval antigen were quantified in an ELISA. Strongyle type eggs were detected in 66.7% of the individual faecal samples. Nematode DNA was amplifiable from 311 samples and *S. vulgaris* and *Strongylus edentatus* were detected in four (1.3%) and 10 (6.3%) of these, respectively, the latter using a novel high-resolution-melt PCR targeting *S. edentatus, Strongylus equinus*, and *Strongylus asini*. On the farm level, prevalence for *Strongylus* spp. by PCR was 12.5%. Applying a conservative cut-off (sensitivity 0.43, specificity 0.96), 21.2% of all serum samples were positive for antibodies against *S. vulgaris* larvae (83.3% prevalence on farm level). Newly developed pyrosequencing assays to analyse putatively benzimidazole resistance associated polymorphisms in codons 167, 198, and 200 of the isotype 1 β-tubulin gene of *S. vulgaris* did not detect such polymorphisms in the four positive samples. Low age and increasing access to pasture were risk factors for egg shedding and seropositivity for *S. vulgaris*. Time since last treatment increased whereas use of moxidectin and ivermectin for the last treatment decreased the risk for strongyle egg shedding. Noteworthy, horses under selective treatment had significantly higher odds to be seropositive for anti-*S. vulgaris* antibodies than horses treated four times per year (odds ratio 4.4). The serological findings suggest that exposure to *S. vulgaris* is considerably higher than expected from direct diagnostic approaches. One potential explanation is the contamination of the environment by a few infected horses, leading to the infection of many horses with larvae that never reach maturity due to regular anthelmintic treatments.

## Introduction

The most common and pathogenic nematode parasites in horses originate from the family Strongylidae and the subfamilies Cyathostominae and Strongylinae, respectively. They differ particularly concerning size and shape of the buccal capsule (1). The Cyathostominae (cyathostomins or small strongyles) show the highest prevalence among all helminths of horses. They do not perform tissue migration during their development in the host although they have intramucosal stages that might also become hypobiotic (2–8). In contrast, the most important members of the Strongylinae (large strongyles), which belong to the genus *Strongylus*, show complex species-specific tissue migrations through different abdominal organs over several months. During these migrations, they can cause severe tissue damage, clinical signs, and can lead to life threatening complications (9, 10).

Previous studies revealed high prevalence of strongyles on German horse farms with 91.6% by Wirtherle et al. (11) in Lower Saxony, 98.7% by Fritzen et al. (12) in North Rhine-Westphalia and 98.4% by Hinney et al. (13) in Brandenburg on the farm level. A modified McMaster method was used in all these studies. Additional coproscopic techniques (combined sedimentation/flotation technique, perianal tape test, sedimentation, and Baermann-Wetzel method) were only performed in the latter study. Kaspar et al. (14) reported a prevalence of 55.3% on the individual horse level in a study testing horses from 91 stables across Germany using a modified McMaster method and combined sedimentation/flotation. In another study using the modified McMaster method, 44.6% of the equines from 192 horse farms across Germany were found to shed strongyle eggs (15). To date, cyathostomins are considered to contribute to the vast majority of the egg shedding, since the prevalence of large strongyles was found to have dropped to very low levels following the introduction of anthelmintics with efficacy against migratory stages of these parasites in the early 1980's (16, 17). Table 1 shows the prevalence of *Strongylus vulgaris* as determined in earlier studies conducted in Germany. Using larval culture and subsequent morphological differentiation of the third stage larvae (L3), prevalences of 0.1–1.3% were observed (13–15, 18, 23).

**Table 1:** Prevalence of *Strongylus vulgaris* in former studies conducted in Germany.

**Region**	**Year^a^**	**Study design**		**Prevalence %**	**Reference**
		**Method**	**HL/FL^b^**		
Nationwide	2013/2014	qPCR	HL	1.9	Kaspar et al. (14)
			FL	10.9	
		Faecal culture, larval identification	HL	1.1	
			FL	4.8	
Nationwide	2012/2013	Faecal culture, larval identification	HL	0.1	Schneider et al. (15)
Southern Germany	2011/2012	Faecal culture, larval identification	HL	1.1	Greite (18)
			FL	5.9	
Brandenburg	2006	Faecal culture, larval identification	FL	0.8	Hinney et al. (13)
North Rhine-Westphalia	2003/2004	Faecal culture, larval identification	FL	1.3	Fritzen et al. (12)
Upper Bavaria	1993/1994	Faecal culture, larval identification	HL	0	Beelitz et al. (19)
	1994/1995		HL	0	Beelitz et al. (20)
Lower Saxony	1995	Post mortem sectio	HL	100	Cirak et al. (21)
Nationwide	1958	Post mortem sectio	HL	79.8	Kiedrowski (22)

a *Sampling year*.

b *HL, horse level; FL, farm level*.

The prepatent period of *S. vulgaris* ranges from six to seven months (24). After oral ingestion of the infective third larval stage (L3), which develops on pasture, L3 undergoes exsheathment and penetrates the mucosa of the caecum and colon (25). The life cycle of *S. vulgaris* comprises an extensive parenteral larval migration. The L3 moult in the mucosa of the large intestine to L4 and starting 2 weeks post-infection begin to migrate through the intestinal arteries and the cranial mesenteric artery. They migrate both on and in the intima of the blood vessels. After about 3 months, another moult to the preadult stage occurs, and another 4–6 weeks later the worms move back with the blood stream towards the intestine (26). The most frequent clinical signs of infection with *S. vulgaris* are fever, lethargy, weight loss, and colic (26–28). The extensive parenteral migration of the larvae in the cranial mesenteric artery and its branches causes endothelial damage, resulting in inflammatory reactions. The endothelial damages lead to the development of thrombi from which emboli result, which end up in the small and large intestinal wall leading to haemorrhagic infarctions. Thickening of the arterial walls occurs (24), leading to impaired blood flow and sometimes severe damage of the arterial wall due to passive dilatation which has been reported to also result in sponge-like so-called “verminous aneurysms” (29–34). In more recent reports clinical infections with *S. vulgaris* have been associated with the appearance of non-strangulating intestinal infarction, leading to peritonitis and mild to severe colic (27, 35).

The larvae of *Strongylus edentatus* migrate to the liver through the intestinal veins. An infection is rarely reported to be associated with clinical signs (36, 37). Like *S. edentatus*, the larvae of *Strongylus equinus* also migrate through the liver, but they also pass through the pancreas and cause inflammation in both organs (38). *Strongylus asini* is described to occur in zebras and donkeys. It is reported to cause an extensive liver pathology. Horses are much less susceptible to *S. asini* than other equine species (39).

The prepatency of the cyatostomins ranges from 1.5 to 3 months (40–42). However, cyathostomin larvae can remain encysted in the caecal or colonic mucosa for 2 years or even longer when the maturation of the early L3 is arrested (7, 43, 44). Cyathostomin infections are associated with the occurrence of milder symptoms such as weight loss. However, synchronised egress of hypobiotic larvae from the mucosa can cause larval cyathostominosis that may lead to severe clinical signs such as weight loss, protein-losing diarrhoea and colic, and even death (43, 45, 46).

Detection of an equine infection with nematodes is often carried out using a coproscopic method. However, it is not possible to reliably distinguish the eggs of Cyathostominae and Strongylinae based on their morphology. Instead a larval culture is required for this purpose, since the L3 can be distinguished microscopically between the two and also to the species level concerning *Strongylus* spp. using details such as the number of mid gut cells (47).

A real-time PCR (48), conventional PCR, and other PCR-based methods (48–51) have been described to identify patent infections with *S. vulgaris* using eggs or larval stages. It was reported that significantly higher numbers of horses were tested *S. vulgaris* positive by real-time PCR and conventional PCR than by morphological examination of L3 from larval cultures (14, 52). Serological detection of infections with cyathostomin species (53) or *S. vulgaris* (54) have been described to be possible using enzyme-linked immunosorbent assays (ELISAs).

Currently, three broad-spectrum anthelmintic classes are commercially available by prescription for nematode control in horses in Germany: Tetrahydropyrimidines (pyrantel pamoate), macrocyclic lactones (MLs, ivermectin, moxidectin), and benzimidazoles (BZs, fenbendazole). They have been used for decades to treat and control equine nematode infections. Encysted, hypobiotic cyathostomin larvae are known to be rather insensitive to currently available anthelmintics but can be affected by treatment with moxidectin (with moderate efficacy) (16) as well as a five-day treatment with fenbendazole, the latter provided the present cyathostomin population has not developed BZ resistance (55, 56).

Anthelmintic resistance (AR) in equine nematodes is widespread (4, 57–59). Concerning strongyles it is primarily the BZ drug class against which cyathostomin populations have developed resistance, but also resistance to pyrantel is prevalent (57, 60–66). Molecular analysis of AR associated genetic changes, for example concerning the respective drug target, promises to allow the detection of AR development at earlier stages than phenotypic tests allow (67). Benzimidazoles inhibit the polymerization of α- and β-tubulin dimers into microtubules (68, 69). Disruption of microtubule polymerisation is supposed to affect the energy metabolism and inhibit the fumarate reductase and the glucose uptake in some nematode species, which leads to the death of the worms (70). Benzimidazole resistance in strongyle nematodes is particularly associated with single-nucleotide polymorphisms (SNPs) in the isotype 1 β tubulin gene. Exchanges in codons 167, 198, or 200 have been described to be associated with BZ resistance (71–78). It has been recorded that the SNP at codon 167 was predominantly associated with BZ resistance in cyathostomins (79).

The increasing emergence of AR of cyathostomins worldwide is a substantial concern in terms of recommendations of appropriate parasite control schemes (80). A surveillance-based approach using faecal egg count analysis (FEC) before anthelmintic treatment has been introduced in several European countries such as Denmark, Sweden, Finland, the Netherlands, and Italy (81). A prescription-only sale of anthelmintic drugs for horses has been implemented with the aim to reduce the risk of AR development. However, no association between presence of *S. vulgaris* and the magnitude of the FEC has been found (82, 83). A reappearance of *S. vulgaris* has been reported in Denmark and Sweden upon the introduction of prescription-only policies (82, 84).

The aim of this article is to provide: (i) an overview about the distribution of nematodes in horses in Berlin and Brandenburg, Germany and (ii) an insight into the occurrence of large strongyles with a focus on *S. vulgaris*. Additional aims were to describe (iii) a real-time PCR with subsequent high-resolution melt (HRM) analysis for species differentiation of large strongyles other than *S. vulgaris* and (iv) to develop pyrosequencing assays for *S. vulgaris* to determine allele frequencies in the BZ resistance associated codons 167, 198, and 200 of the β-tubulin isotype 1 gene.

## Materials and Methods

### Ethics Approval

All experimental procedures were conducted in accordance with European (EU directive 2010/63/EU) and German (“Tierschutzgesetz”) laws and were approved by local authorities (Landesamt für Gesundheit und Soziales, Berlin, registration no.: Reg 0059/17).

### Study Design and Location

Serum, saliva, and faecal samples were collected between May 2017 and January 2018, and all samples were obtained from naturally infected horses. In this study, strategic deworming management was defined as a periodically repeated anthelmintic treatment applied to all or the majority of horses on a farm carried out without prior testing to identify parasitic infections. Selective deworming was defined as an anthelmintic treatment on individual horses according to a predetermined diagnosis, most commonly based on FEC. Each farm was asked to respond to a questionnaire with questions about the current parasite control management as well as pasture and hygiene management (Supplementary Questionnaire 1) (85). Data regarding eggs of Anoplocephalidae and antibodies against equine tapeworms in serum and/or saliva in the same sample set have been published previously (85).

### Coproscopic Analysis

Mini-FLOTAC (University of Naples Federico II, FLOTAC® Group, Naples, Italy) counting chambers (86, 87) were used to determine FEC by processing a 5 g faecal sample with the Fill-FLOTAC device (University of Naples Federico II, FLOTAC® Group, Naples, Italy), as previously described by Noel et al. (88), with 45 ml of saturated saline solution (specific gravity 1.2) being added. In addition, a double centrifugation/combination sedimentation flotation method was used according to the technique published by Rehbein et al. (89). Per faecal sample, 15 g faeces were used. After the first centrifugation step, the pellet was floated using a concentrated sucrose solution (specific gravity 1.26). Material from the surface of the solution was transferred with a horizontal wire loop to a glass slide. Eggs were counted and the egg shedding intensity was categorised as follows: 0: no eggs found; 1: 1–10 eggs per slide; 2: 11–40 eggs per slide; 3: ≥ 41 eggs per slide.

### Strongyle Larval Cultures and DNA-Extraction

Strongyle larval cultures and DNA-extraction were carried out with samples that were coproscopically positive for strongyle type eggs. If possible, ~200 g (at least 50 g) of unrefrigerated fresh faecal sample material per individual animal were used for cultivation of larvae. The faecal samples were loosened manually and placed in glass jars (500 g capacity with plastic screw cap). Sawdust was added to the low viscosity samples. The plastic screw cap of the jars was placed lightly on the glass container. The samples were placed into an incubator cabinet at 25°C and a relative humidity of about 80% for 10–14 days. For harvesting of L3, jars were completely filled with tap water, a Petri dish was placed on its top and both were rapidly inverted. After incubation at room temperature for 12–16 h larvae were harvested from the water in the petri dish surrounding the glass jar.

The extraction of genomic DNA from all L3 collected for individual horses was performed using the NucleoSpin® Soil Kit (Macherey-Nagel) with buffer SL1 according to the manufacturer's instructions. DNA was eluted with 30 μl elution buffer. Deviating from the protocol, the SpeedMill P12 from Analytik Jena AG (Jena, Germany) was used to homogenise the samples.

### Polymerase Chain Reactions

#### PCR for the Detection of Pan-Nematode-DNA

Presence of amplifiable nematode DNA from strongyle L3 was verified using a pan-nematode PCR targeting the 28S rRNA gene as described by Demeler et al. (90). PCR reactions contained 250 nM of each primer, 200 μM dNTPs,0.02 U/μl Phusion Hot Start II High-Fidelity DNA Polymerase (Thermo and 2.0 μl template DNA in 20 μl 1× Phusion HF buffer. As positive control, plasmid DNA (80 copies) containing the corresponding 28S rRNA fragment from *Teladorsagia circumcincta* cloned in pCR®2.1-TOPO was used. After an initial denaturation at 98°C for 30 s, 40 cycles of 98°C for 10 s, 55°C for 30 s, and 72°C for 30 s were performed. A final extension was carried out at 72°C for 5 min. If no PCR product could be detected when 2 μl of the product were analysed on 1.5% agarose gels stained with GrGreen (Labgene, Châtel-Saint-Denis, Switzerland), possibly due to the presence of PCR inhibitors, the template DNA was diluted 1:5 with DEPC-treated water and the PCR was repeated.

#### *Strongylus vulgaris*-Specific Real-Time PCR

The DNA samples positive in the pan-nematode PCR were further analysed using an *S. vulgaris* specific real-time PCR targeting a partial ITS-2 region initially described by Nielsen et al. (48) as modified by Gehlen et al. (91). As positive control, plasmid DNA containing the ITS-2 amplicon from *S. vulgaris* in the vector pSC-B-amp/kan (Aglient Technologies, Waldbronn, Germany) was used. In each run, three different dilutions of the positive control (500, 50, and 5 copies) were analysed in parallel with the field samples. PCR reactions contained 250 nM of each primer (Supplementary Table 1) in 20 μl 1× GoTaq® qPCR Master Mix and 1 μl template DNA. After an initial denaturation at 95°C for 2 min, 50 cycles consisting of denaturation at 95°C for 15 s, annealing at 61°C for 30 s and elongation at 72°C for 30 s were conducted. The subsequent melting curve analysis consisted of a rise in temperature from 68°C−95°C in 0.5°C increments for 5 s with fluorescence monitoring in each step. All samples positive for *S. vulgaris* according to amplification and melting curves were also analysed by gel electrophoresis on 2.0% agarose gels to control the size of the PCR products. Finally, PCR products were purified using the DNA Clean & Concentrator®-5 purification kit (Zymo Research, Freiburg, Germany) and sent to LGC Genomics (Berlin, Germany) for Sanger sequencing. The determined DNA sequences were compared to the NCBI database using Blastn (92).

#### Real-Time PCR and High-Resolution-Melt PCR Analysis for Species Differentiation of *Strongylus edentatus, Strongylus equinus*, and *Strongylus asini*

In order to identify samples positive for the three other members of the genus *Strongylus*, a single PCR suitable for amplifying partial *S. edentatus, S. equinus*, and *S. asini* ITS-2 fragments from *Equus* spp. derived samples was developed as a real-time PCR followed by a high-resolution-melt PCR analysis. Under the optimised PCR conditions, the primer pair that was used did not amplify *S. vulgaris* DNA from a plasmid containing the *S. vulgaris* ITS-2 region as insert. Plasmids for *S. equinus* and *S. asini* to be used as positive controls were ordered from Shanghai ShineGene Molecular Bio-Technologies, Inc. and contained the ITS-2 according to the GenBank accession number X77808.1 for *S. equinus* and X99345.1 for *S. asini*. For the controls of *S. vulgaris* and *S. edentatus*, the amplified ITS-2 sequence was inserted in the cloning vector pSC-B-amp/kan. In each PCR run, positive controls containing plasmid DNA corresponding to the amplicons from *S. edentatus, S. equinus*, and *S. asini* as templates (in duplicate with 500 copies per reaction) were run, in parallel to be used as references for melting curves. PCR reactions contained 500 nM of each primer (Supplementary Table 1) in 20 μl 1× GoTaq® qPCR Master Mix and 1 μl template DNA. The PCR scheme consisted of an initial denaturation at 94°C for 2 min followed by 50 cycles of denaturation at 94°C for 10 s, annealing at 63°C for 30 s and elongation at 72°C for 30 s. Fluorescence was measured throughout the elongation phase. Subsequently, an HRM analysis was performed by raising the temperature from 65–98°C in 0.1°C increments with each step lasting for 10 s each and fluorescence read at each step. Melting curves of PCR products were assigned to a large strongyle species using the Precision Melt Analysis Software 1.3 (Bio-Rad). All positive samples were validated by Sanger sequencing of the PCR product as described above.

### Pyrosequencing of Isotype 1 β-Tubulin Polymorphisms

A partial *S. vulgaris* isotype-1 β-tubulin gene sequence was obtained using primers obtained from isotype-1 β-tubulin gene fragments in the *S. vulgaris* genome database (WormBase ParaSite PRJEB531) (93, 94). One pyrosequencing assay for the analysis of the BZ resistance associated amino acid exchange F167Y and a second for E198A and F200Y of the isotype-1 β-tubulin gene was designed based on the *S. vulgaris* isotype β-tubulin isotype 1 sequences using the Pyromark Assay Design Software version 2.0 (Qiagen, Hilden).

Plasmid DNAs containing the PCR amplicons with the susceptible genotypes were obtained by PCR using DNA of adult *S. vulgaris* specimens stored in the parasite archive of the Institute for Parasitology and Tropical Veterinary Medicine at −80°C. Plasmids with inserts encoding the resistance-associated genotypes were synthesised by General Biosystems, Inc. (Morrisville, USA). Target sequences were amplified using a PCR covering codon 167 and another covering codons 198/200, both using a biotinylated reverse primer (Supplementary Table 2). PCRs contained 200 μM dNTPs, 250 nM of each primer, 0.02 U/μl Phusion Hot Start II High-Fidelity DNA Polymerase and 2 μl template DNA in 50 μl 1× HF buffer. As template DNA, either defined mixtures of plasmid DNA with different genotypes at codon positions 167, 198, or 200 were used or DNA extracted from the L3 pool collected from individual horses. Amplification was initiated by denaturation at 98°C for 1 min followed by 50 cycles of 98°C for 15 s, 62°C for 30 s, and 72°C for 20 s. Finally, reactions were incubated at 72°C for 5 min. PCR products were analysed on 2% agarose gels. Then, pyrosequencing was conducted using the Qiagen PyroMark® Q24 System according to the manufacturer's instructions. The only deviation from the protocol was to use a larger volume (40 μl) of PCR product to be immobilised on Streptavidin-Agarose beads (Sigma-Aldrich, Taufkirchen, Germany).

Initially, pyrosequencing assays were evaluated using defined mixtures of genotypes associated with susceptibility and resistance by mixing plasmid DNAs in the ratios 0/100%, 10/90%, 20/80%, …, 90/10%, 100/0%. Pyrograms were analysed using the Pyromark Q24 2.0.8 software.

### *Strongylus vulgaris* ELISA

Frozen serum samples were sent to the M.H. Gluck Equine Research Centre, Department of Veterinary Science, University of Kentucky, Lexington, KY, USA and tested with an indirect ELISA using a recombinant SvSXP (rSvSXP) protein expressed by migrating *S. vulgaris* larvae as antigen (54).

Based on the optical density of positive and negative controls and sample wells on the same plate, an optical density ratio (ODR) was calculated according to the formula:


$$\begin{array}{l} \text{ODR}[\%]=[\text{OD}(\text{Sample})\text{-OD}(\text{NC})]\\ \text{ /}{\left[\text{OD}(\text{PC})\text{-OD}(\text{NC})\right]}^{*}\text{100} \end{array}$$

(OD: optical density, NC: negative control, PC: positive control).

The cut-off value of an ODR of 13.5% (sensitivity 65.5% and specificity 82.1%) as established by Andersen et al. (54) was not applied to evaluate the results of the rSvSXP ELISA in the present study. Instead, a more conservative approach was chosen to evaluate the results: Cut-off values of ODR of 25% (sensitivity 0.50, specificity 0.86) and 30% (sensitivity 0.43, specificity 0.96) were used to identify potentially positive samples.

### Statistical Analyses

Data were entered into Microsoft Excel spreadsheets. Pearson correlation analyses and all graphs were created using GraphPad Prism® version 5.03. All other statistical analyses were performed with R 3.4.4 in RStudio version 1.1.456 for Windows. For percentage of positive animals, 95% CIs were calculated as Wilson score intervals using the binom.wilson function in the prevalence 0.5–10.1 package.

To estimate the inter-rater agreement to compare the faecal analysis methods the Cohen's kappa coefficients were calculated using the cohen.kappa() function in R.

For the evaluation of pyrosequencing-based analysis of putatively BZ resistance associated polymorphisms in the isotype-1 β-tubulin gene of *S. vulgaris*, observed codon frequencies were plotted against calculated input frequencies. Linear regression plots with 95% confidence bands were calculated and plotted.

For identification of risk factors for strongyle egg shedding status and for being detected positive for *S. vulgaris* by serology, logistic regression models were calculated using the glm() function in R. Initially, all explanatory variables that were considered to likely affect the odds of a horse to be positive for strongyle eggs or antibodies against *S. vulgaris* were included. Backward elimination of variables was performed using the drop1() function eliminating always the variable that resulted in the strongest decrease in the Akaike Information Criterion (AIC). Odds ratios and 95% CIs were obtained from parameter estimates and errors by calculating the exponential value of the results and calculating the 95% CIs using the profiling approach as implemented in the confint() function of the MASS package version 7.3–54. A set of pseudo R^2^ values was calculated for the final logistic regression model using the PseudoR^2^ function from the DescTools 0.99.27 package.

## Results

### Study Population and Collection of Field Samples

Faeces and serum samples were collected from 484 domestic horses from 48 yards. The study was planned to include horses that had not received anthelmintic treatment for at least 6 weeks. However, a shorter period of time elapsed between the last deworming and sampling was subsequently reported for 13 horses. For one horse, no conclusive information was available regarding the last deworming. Table 2 shows a modified version of Table 2 from Jürgenschellert et al. (85) containing general data of the study population relevant for the data presented here. The median time between the last anthelmintic treatment and sampling was 14.4 weeks. Access to pasture was available for 96.7% of the horses (with at least seasonal access). Most of the farms followed a strategic deworming approach, therefore 91.3% of the sampled equines had received anthelmintics on a regular basis.

**Table 2:** General data on the study population (484 horses from 48 farms).

**Parameter**	**Value**
Age of the sampled horses [years; median (range)]	12.0 (0.8–34.0)
Mares (%)	50.2
Geldings (%)	46.7
Stallions (%)	3.1
Faecal samples (%)	100
Serum samples (%)	99.4
Last treatment	
Ivermectin + Praziquantel (%)	7.2
Ivermectin (%)	42.4
Moxidectin (%)	4.1
Doramectin (%)	1.4
Fenbendazole (%)	4.3
Pyrantel (%)	38.2
n.a.^a^	2.3
Period between last anthelmintic treatment and sampling [weeks; median (range)]	14.4 (1.4–100.0)
Horses sampled per farm [number; median (range)]	10 (4–17)
Pasture access (%)	96.7
Unlimited (%)	28.9
Limited (%)	67.8
Pasture area/horse (ha) (median, range)	0.5 (0.1–2.0)
Total number of horses kept on farm [median (range)]	30 (6–110)
Foals present (%)	39.6
Total number of foals kept on farm [median (range)]	0 (0–10)
Treatment schedule (annual)	
Selective (%)	8.7
Dewormed 1–2.5 times/year (< 3 times) (%)	32.8
Dewormed 3–3.5 times/year (<4 times) (%)	39.6
Dewormed 4 times/year (%)	18.9

a *Not available*.

### Faecal Sample Analyses

Strongyle eggs were found in 66.7% (95% CI 62.4–70.8%) of the samples. The FEC of the strongyle type egg positive faecal samples ranged from 5–2,935 epg. An epg > 200 was recorded in 136 samples (28.1%; 95% CI 24.3–32.3%). Eggs of *Oxyuris equi, Anoplocephala* spp. and *Parascaris* spp. were identified in 1.2% (95% CI 6–2.7%),0.6% (95% CI 2–1.8%) and 0.4% (95% CI 1–1.5%) of the faecal samples, respectively. The results of the two coproscopic methods employed in parallel are shown in Table 3. Strongyle eggs were detected in 64.3% (95% CI 59.9–68.4%) of the faecal samples with the Mini-FLOTAC and in 57.4% (95% CI 53.0–61.8%) with the combined sedimentation-flotation method.

**Table 3:** Percentage of 484 equine faecal samples tested positive using different FEC methods.

**Method**	**Strongyle egg [%] (95% CI^a^)**	***Anoplocephala* spp. [%] (95% CI^a^)**	***Oxyuris equi* [%] (95% CI^a^)**	***Parascaris* spp. [%] (95% CI^a^)**
Sedimentation / flotation	57.4 (53.0–61.8)	0.6 (0.2–1.8)	0.8 (0.3–2.1)	0 (0.0–0.6)
Mini-FLOTAC	64.3 (59.9–68.4)	0.6 (0.2–1.8)	1.2 (0.6–2.7)	0.4 (0.1–1.5)
Both coproscopic methods	66.7 (62.4–70.8)	0.6 (0.2–1.8)	1.2 (0.6–2.7)	0.4 (0.1–1.5)
Farm level	97.9 (89.1–99.6)	6.3 (2.1–16.8)	12.5 (5.9–24.7)	2.1 (0.4–10.9)

a *CI, confidence interval*.

**Figure 1:**
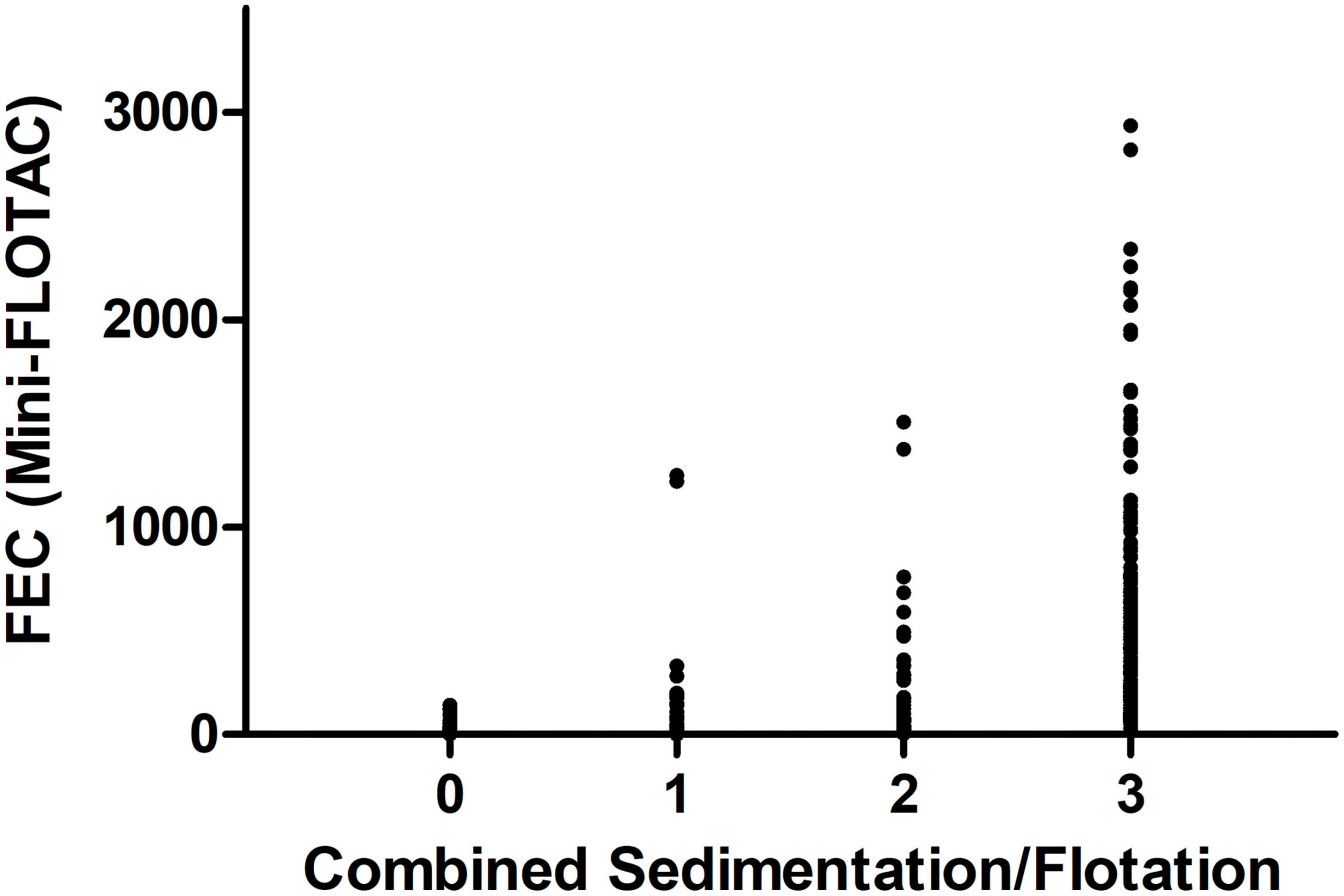
Comparison of faecal analyses methods, referring to counting results of strongyle type eggs of 484 faecal samples. Results of the combined sedimentation/flotation methods are plotted as classes (0: no eggs found; 1: 1–10 eggs per slide; 2: 11–40 eggs per slide; 3: ≥ 41 eggs per slide) vs. eggs per gram faeces (epg) as determined using Mini-FLOTAC.

The faecal samples of 161 horses did not show strongyle type eggs in any of the coproscopic analyses. In total, 266 samples were positive using both methods, while 45 were only positive using Mini-FLOTAC, and 12 samples were only positive using sedimentation/flotation. To estimate inter-rater agreement from these data, the Cohen's kappa coefficient was calculated to be κ = 0.75 (95% CI 69–0.81). In Figure 1, the quantitative results of the two faecal analyses methods are plotted against each other. Despite strong overlap of FECs obtained by Mini-FLOTAC between the different semi-quantitative categories obtained by sedimentation/flotation, the Spearman's rank correlation coefficient indicated a significantly higher FEC in higher classes of the sedimentation/flotation method (ρ = 0.8714; *p* <0.0001).

Larval cultures and subsequent DNA extractions from purified L3 were carried out for 323 individual faecal samples that were tested positive for strongyle type eggs with Mini-FLOTAC or the combined sedimentation-flotation method.

### Frequency of *Strongylus spp*. in DNA From Larval Cultures

#### Detection of Nematode DNA Using a PCR Targeting the 28S rRNA Gene

The 28S rRNA pan-nematode PCR was performed on all 323 samples that showed a strongyle type egg. The PCR result indicated the presence of amplifiable nematode DNA in 259 samples. The PCR was repeated on 64 samples with 1:5 diluted templates. Out of these runs, 12 samples remained negative, while a PCR product was obtained for an additional 52 samples. In total, the 28S PCR was able to detect nematode DNA in 311 samples. Only these samples were further investigated using PCR targeting *S. vulgaris* and *S. edentatus*/*equinus*, respectively.

#### Detection of *Strongylus vulgaris* DNA by Real-Time PCR

**Figure 2:**
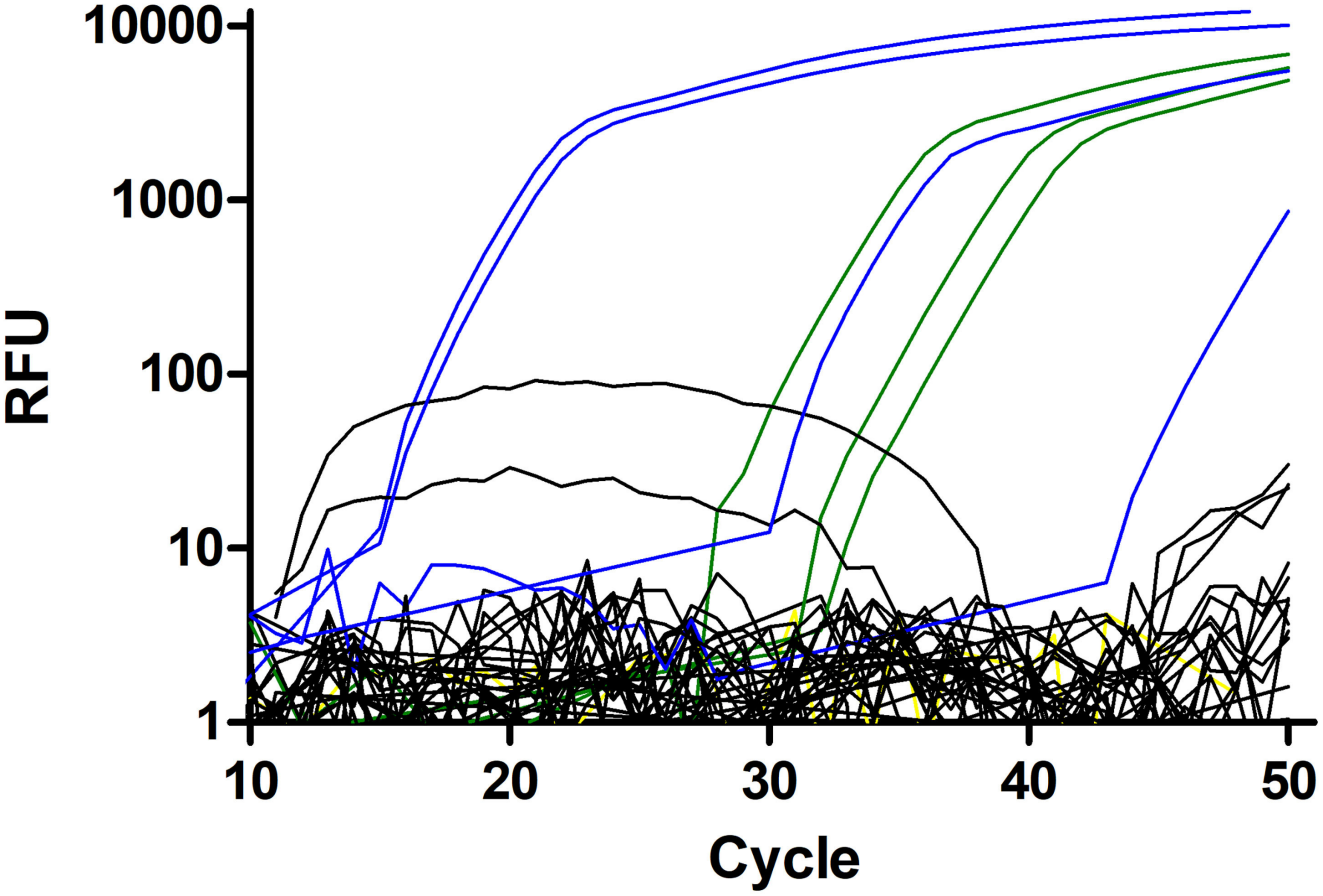
Amplification plots for the *Strongylus vulgaris* real-time PCR. Plasmid DNAs with 500, 50, and 5 copies were used as positive control (green). The single negative control is shown in yellow but mostly hidden by the black curves. Field samples considered to be negative are shown in black and samples scored as positive are in blue. All positive samples, including the one with a very high Cq value were repeatedly tested positive and identity of all PCR products was confirmed by Sanger sequencing.

The PCR specific for *S. vulgaris* was positive for four samples (1.3% of the horses for which nematode DNA was amplifiable; 95% CI.5–3.3%) (Figure 2). Positive samples were confirmed by sequencing purified PCR products followed by comparison with GenBank entries. After removing low quality sequence regions at the beginning and the end of the chromatograms, Blastn searches against GenBank revealed 99–100% identity to the GenBank entries KT2506171.1 and KT250621.1. On the farm level, 3 out of 48 farms (6.3%, 95% CI 2.1–16.8%) were positive for *S. vulgaris*.

#### Detection and Identification of *Strongylus edentatus* by Real-Time PCR and Differentiation Using High-Resolution-Melt Analysis

**Figure 3:**
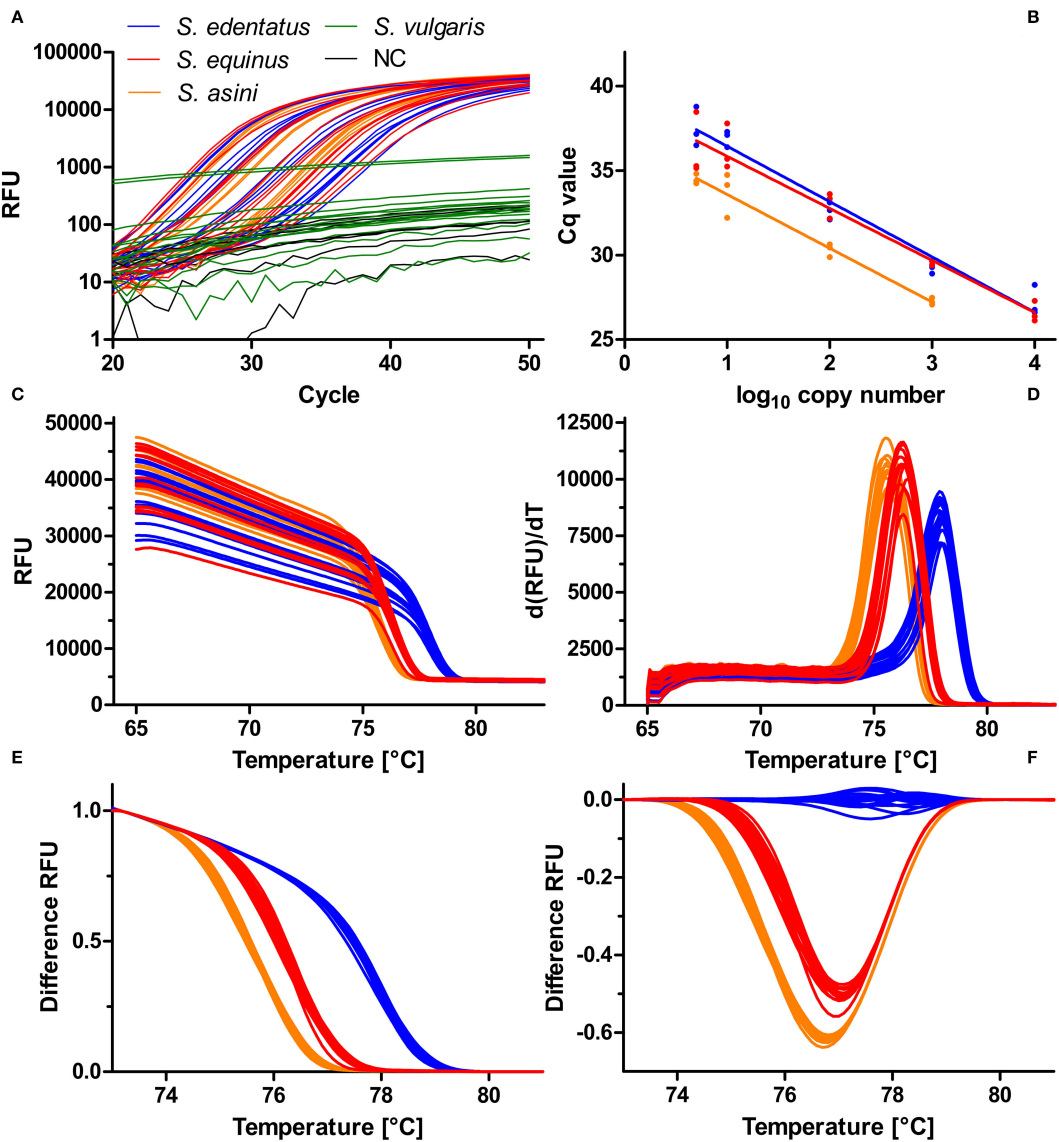
Establishment of a high-resolution melt PCR to distinguish *Strongylus edentatus, Strongylus equinus*, and *Strongylus asini*. PCR. The amplification plots show that presence of 10,000–5 copies (*S. edentatus, S. equinus*) or 1,000–5 copies (*S. asini*) ITS-2 plasmid resulted exponential amplification while there was no exponential increase of relative fluorescence units (RFU) in the presence of 10,000 to 5 copies of *S. vulgaris* ITS-2 DNA or in the absence of any template (NC). **(A)**. Using the data from **(A)**, the quantification cycles (Cq) were plotted against the log_10_ transformed copy numbers and linear regressions were calculated **(B)**. There were no significant differences between slopes (*p* = 0.760). Raw melting curves **(C)** and their first derivative dRFU/dT **(D)** were recorded between 65°C and 98°C. Normalisation of the melting curves between 73.1 and 79.9°C resulted in clear separation of the melting curves **(E)**, which was also visible in the difference plot **(F)**, in which the mean normalised fluorescence for *S. edentatus* was subtracted from all individual plots.

**Figure 4:**
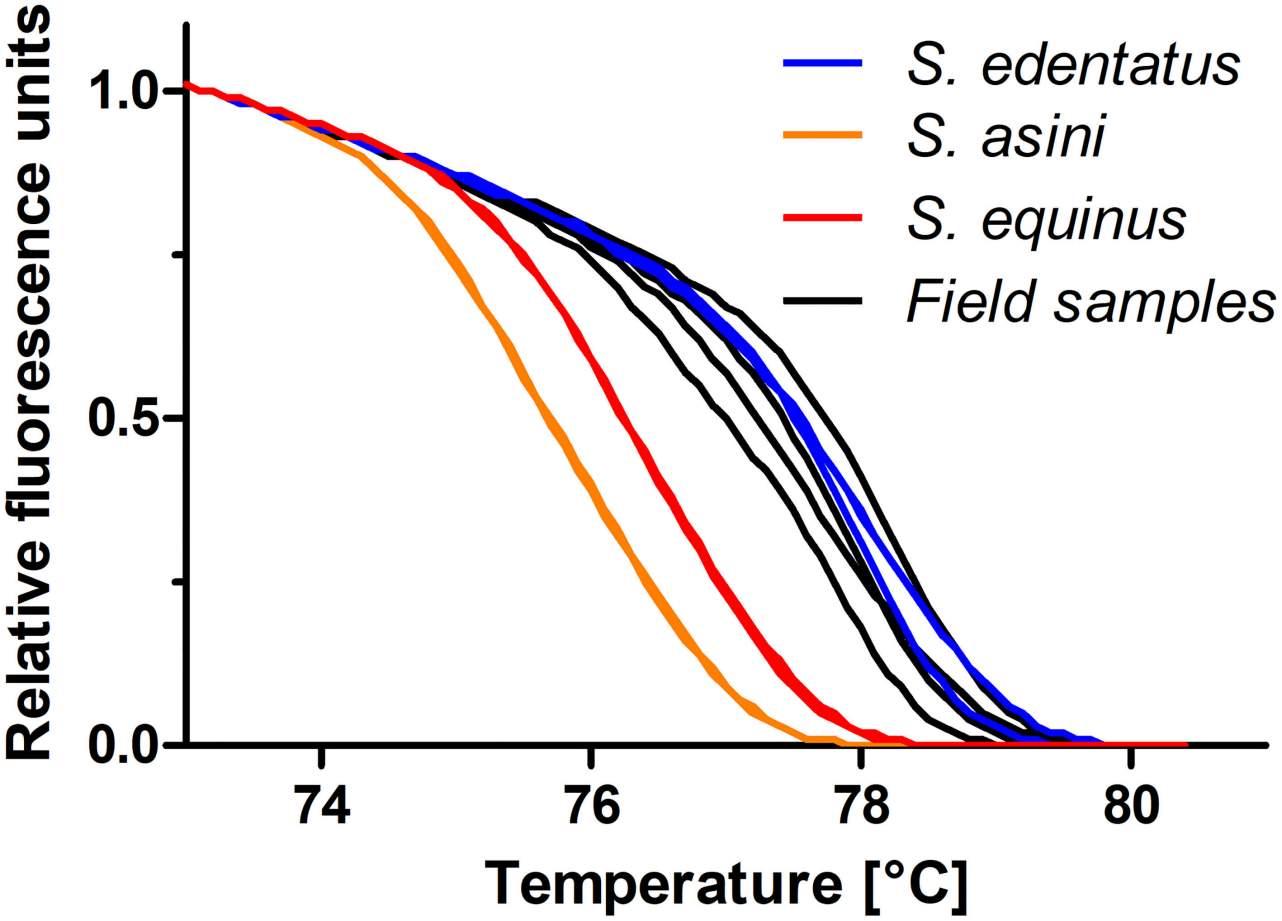
High-resolution melting curves for DNA from four field samples compared to standards containing 500 copies of the ITS-2 of *S. edentatus, S. equinus*, and *S. asini*. All positive samples were assigned to *S. edentatus*, which was confirmed by Sanger sequencing of the PCR products.

This diagnostic PCR was newly established and evaluated using templates containing the amplicon as insert in plasmids. Primers were designed to amplify the three *Strongylus* species *S. edentatus, S. equinus*, and *S. asini* but not *S. vulgaris*. The representative amplification plots depicted in Figure 3A using 10,000 to 5 copies of the plasmid DNA as template show that there was no cross-reactivity with *S. vulgaris* ITS-2 as template. To estimate efficacies, Cq values (y-axis) were plotted against log_10_ transformed copy numbers on the x-axis (Figure 3B). Linear regressions were calculated and tested for differences in slopes in GraphPad. Slopes were −3.273 (efficacy = 111.2%), −3.079 (efficacy = 102.6%) and −3.181 (efficacy = 106.2%) and there was no significant difference in slopes (*p* = 0.760). The raw melting curves and their first derivative are shown in Figures 3C,D. These suggest some degree of differentiation but there was also considerable overlap of the curves. However, normalisation of the curves between 73.1 and 79.9°C resulted in three clearly differentiated clusters of melting curves (Figure 3E) that could also be visualised as different clusters in a difference plot by subtracting the relative fluorescence units (RFUs) of the mean for *S. edentatus* from all individual samples (Figure 3F). In combination with the *S. vulgaris* specific real-time PCR (48), a clear diagnosis of all four large strongyle species is possible combining these two assays. Melting curves for field samples positive for *S. edentatus* are shown in Figure 4.

The results of the real-time PCRs with subsequent HRM analyses indicated that *S. edentatus* was present in 10 equine samples, i.e., 3.2% of the samples with amplifiable nematode DNA, 95% CI 1.8–5.8%). All positive samples were confirmed using Sanger sequencing of PCR products. After removing chromatogram regions with low sequence quality at the beginning and end of sequencing data, all sequences were more than 99% identical to *S. edentatus* such as KP693438.1 or X77807.1.

These 10 individual horses originated from four of the participating farms revealing a prevalence on the farm level of 8.3% (95% CI 3.3–19.6%). There was no amplification of the rRNA gene of *S. equinus* detected from field samples. One of the horses was positive for both, *S. vulgaris* and *S. edentatus*, resulting in a 3.5% frequency of *Strongylus* spp. (95% CI 2.0–6.2%) among the 311 horses for which the 18S nematode PCR was successful. On the farm level, 6 out of 48 positive farms resulted in a prevalence of 12.5% (95% CI 5.9–24.7%).

### Absence of Benzimidazole Resistance Associated Polymorphisms

**Figure 5:**
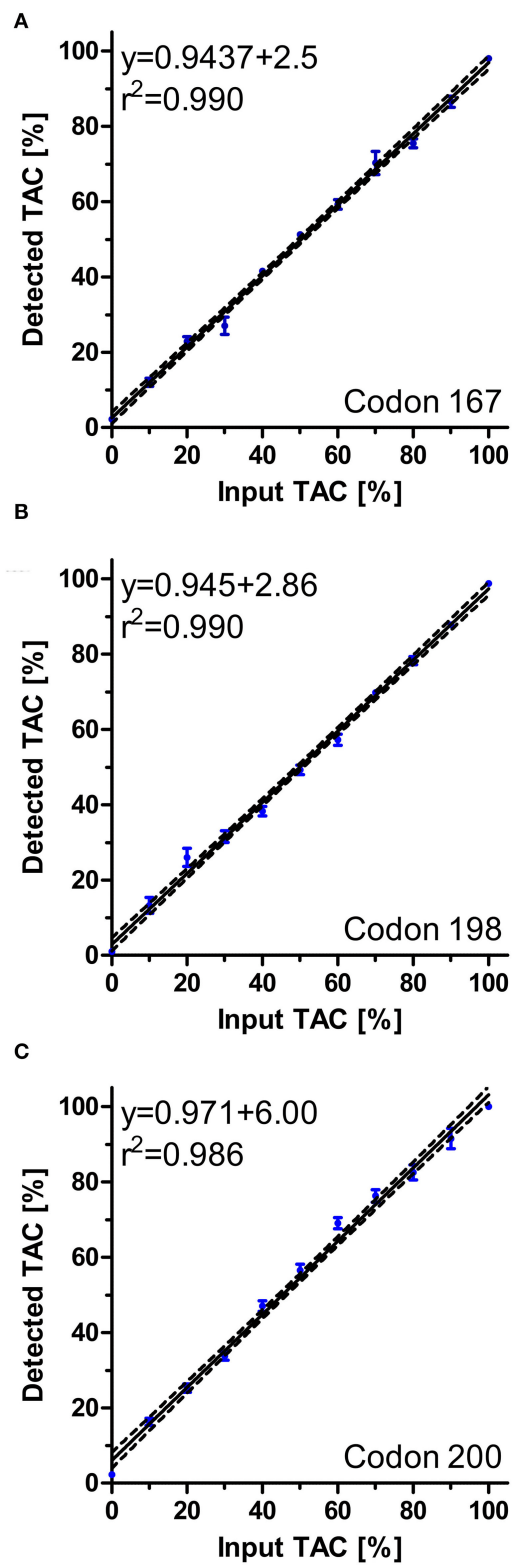
Regression analysis of pyrosequencing assays for *S. vulgaris* isotype 1 β-tubulin for polymorphisms F167Y **(A)**, E198A **(B)** and F200Y **(C)**. Artificial mixtures of plasmids were prepared and analysed using pyrosequencing. For each mixture, four to six replicates were used and included in the analyses. Regression plots with 95% confidence bands as calculated by Pearson regression are shown. Raw data are shown as circles (dots) ± SEM.

The newly designed pyrosequencing assays for the polymorphisms F167Y, E198A, and F200Y of the isotype 1 β-tubulin gene were first evaluated using artificial mixtures of plasmids containing either the wild-type or the variant associated with BZ resistance. Figure 5 shows that for all three assays an excellent agreement between the percentage of resistance associated allele spiked into the template and the measured frequency was obtained. The coefficients of determination were all between 0.986 and 0.990. At an allele frequency of 0% for the resistance associated alleles, means plus 2 SD were calculated to be 5.1, 2.6, and 5.3% for codons 167, 198, and 200, respectively. Thus, a technical background, a conservative cut-off of 10% was therefore chosen to avoid claiming of detection of resistance allele due to technical background in field samples.

All three assays were applied to the four samples that were *S. vulgaris* positive in the real-time PCR. For codons 167 and 198, all pyrosequencing runs revealed frequencies below 5% that were considered technical background. For codon 200, the observed frequency increased to 10–14% but signal intensity was in general very low. All PCR products obtained with these PCRs were also analysed by Sanger sequencing. The obtained sequences completely corresponded to the expected *S. vulgaris* sequence suggesting that the β-tubulin PCRs used to generate the templates for pyrosequencing did not cross-react with the corresponding genes of other Strongylidae present in the field samples. Regarding codon 198, no peaks corresponding to the BZ resistance associated isotype 1 β-tubulin alleles were found.

### Antibodies Against a Recombinant SvSXP *Strongylus vulgaris* Antigen

Results for anti-*S. vulgaris* IgG were obtained for 481 horses, since it was not possible to collect a blood sample from three horses. The ODRs ranged between −5.3 and 114.4% (median 10.1, mean 17.8 ± 22.52). The original cut-off proposed by Andersen et al. (54) was 13.47% ODR, classifying 39.9% of the samples as positive. Since this was an unexpectedly high value and the reported specificity for this cut-off was only 82.1% (54), two other cut-offs were used to identify samples that were considered positive. If the ODR cut-off was set to 30%, 21.2% (95% CI 17.9–25.1%) of the field samples would be considered serologically positive. Under these assumptions, the seroprevalence on farm level would be 83.3% (95% CI 70.4–91.3). According to Andersen et al. (54), a cut-off value of 30% corresponds to a sensitivity of 43% and a specificity of 96%. Lowering the cut-off value to 25% ODR, then even 24.3% (95% CI 20.7–28.4) of the samples would be considered serologically positive. The seroprevalence at farm level would be even 91.7% (95% CI 80.5–96.7). At this cut-off value of 25% ODR, a sensitivity of 50% and a specificity of 86% were calculated by Andersen et al. (54).

### Correspondence Between PCR and ELISA Data

The ODR values for the rSvSXP ELISA (54) of the horses tested positive for *S. vulgaris* in the qPCR ranged from 16.9 to 35.6% (Table 4). Focusing on the three farms where *S. vulgaris* was detected by real-time PCR, the seroprevalences at farm level were 10% (95% CI 1.79–40.42), 12.5% (95% CI 2.24–47.09), and 70% (95% CI 39.68–89.22) – independently of the cut-off value used.

**Table 4:** *Strongylus vulgaris* and *Strongylus edentatus* positive samples.

**Farm ID/Horse ID**	***S. edentatus* (PCR)**	***S. vulgaris* (PCR)**	***S. vulgaris* serum (ODR)**	**FEC (epg)^a^**	**Combined sedimentation-flotation^b^**
01.04	Positive	Negative	1.40	205	3
01.06	Positive	Negative	14.66	15	1
02.02	Positive	Negative	12.93	20	0
02.10	Positive	Negative	6.11	0	1
03.01	Positive	Negative	18.13	20	0
03.04	Positive	Negative	21.31	35	1
03.05	Positive	Negative	82.82	25	0
03.07	Positive	Negative	35.15	25	1
06.08	Positive	Negative	24.40	1,490	3
06.10	Positive	Positive	16.88	1,370	3
14.05	Negative	Positive	33.47	685	3
18.05	Negative	Positive	35.62	425	3
18.07	Negative	Positive	16.93	5	0

a *FEC, faecal egg counts; epg, eggs per gram faeces*.

b *Number of strongyle type eggs per complete microscope slide: 1: 1–10 eggs per microscope slide; 2: 11–40 eggs per microscope slide; 3: ≥ 41 eggs per microscope slide*.

Table 4 provides details of the FEC of the horses that were tested positive for *S. edentatus* or *S. vulgaris* by real-time PCR. One horse (ID 06.10) tested positive for both large strongyle species that were identified in this study. In the faecal analysis for these horses, strongyle type eggs were found in at least one but not always both of the methods in each case. In the majority of *Strongylus* spp. cases (8/13), the epg value as determined by Mini-FLOTAC was below 200.

### Risk Factor Analysis for Strongyle Egg Shedding

**Figure 6:**
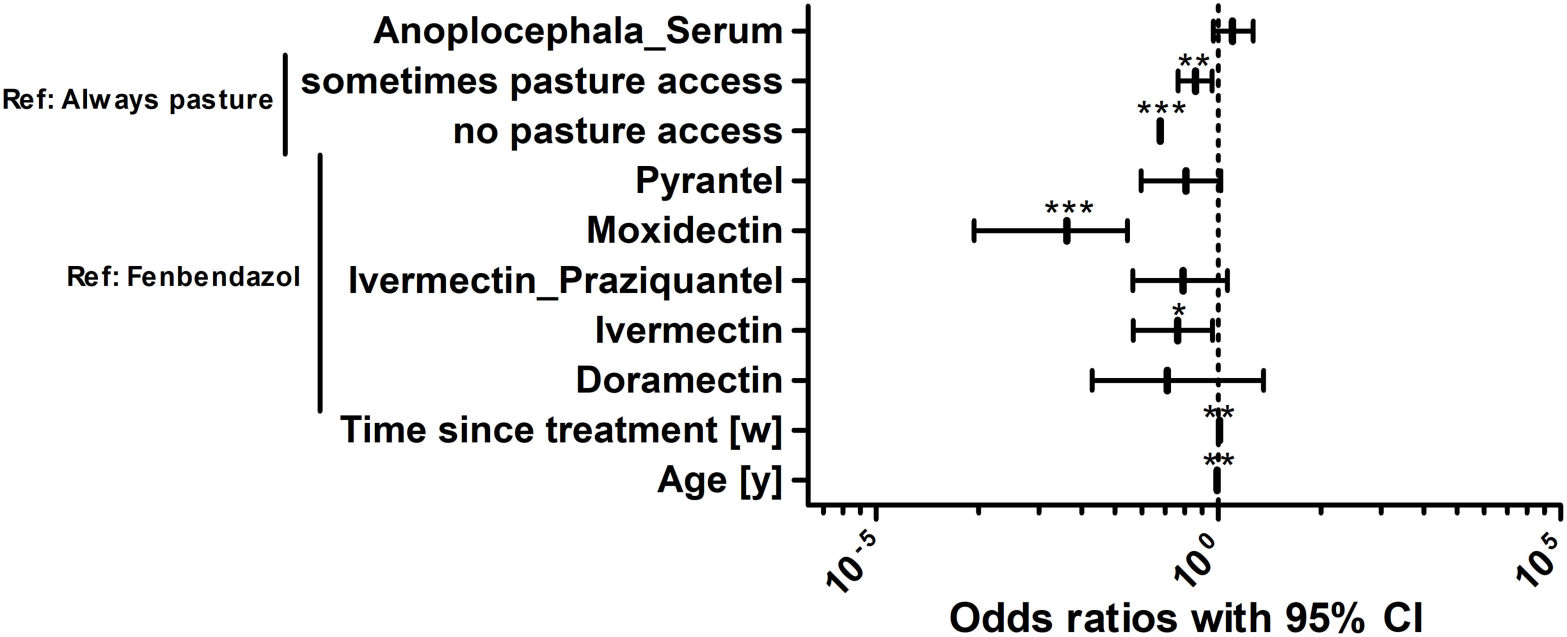
Risk factor analysis for the odds of horses in the study population to be positive for strongyle eggs. Risk factors for the odds to be positive or negative for strongyle eggs were determined using logistic regression analysis. Anoplocephala_Serum, positive in the *Anoplocephala* serum ELISA; Drugs refer to the drug used for the last treatment; Time since treatment represents the time since the last anthelmintic treatment in weeks [w]. The age refers to the age of the animals in years [y]. The age of horses was rounded into whole years. For foals and yearlings, the age was converted from months to years by dividing the month measurement by the conversion ratio 12. ***, *p* < 0.001; **, *p* < 0.01; *, *p* < 0.05.

To identify risk factors that increased the odds of an individual horse in the study population to be positive for strongyle eggs, a logistic regression analysis was performed. A positive finding in this context refers to an identification of at least one strongyle type egg in Mini-FLOTAC and/or in combined sedimentation/flotation. In Figure 6, the odds ratios of different explanatory variables are plotted together with their 95% CIs as forest plots. The variables anthelmintic treatment schedule, total number of horses, the presence of foals on a farm, the pasture area for each horse, whether the horses were periodically standing on different sections of the pasture and whether manure piles were regularly removed from pastures were eliminated from the final model during model optimisation. Further details on the model are given in Table 5. The variable “pasture access”, “limited access” and “no access” to pasture were protective when compared to “continuous access” during grazing season (*p* ρ 0.01). A positive serological *Anoplocephala* spp. ELISA as reported recently for this sample set (85) was in tendency also associated with higher odds to shed strongyle type eggs, but it was not significant. An increased time period between the last anthelmintic treatment and sampling time was also associated with higher odds (*p* < 0.01), i.e., a 1.046 fold increase in odds per week. Regarding the anthelmintic that was used for the last deworming, the use of fenbendazole was associated with higher odds to be positive compared to any other anthelmintic used for the last treatment but the differences were only significant for moxidectin (*p* < 0.001) and ivermectin (*p* < 0.05). The age was significantly associated with lower odds of detecting strongyle eggs in the faeces. With each additional year of age, the odds to be positive for strongyle eggs decreased 0.961 fold (*p* < 0.01). The pseudo-R^2^ according to McFadden (0.16) and Nagelkerke (0.27) can be considered acceptable and show that the model is an improvement over the null model.

**Table 5:** Final logistic regression model to identify risk factors explaining positivity for strongyle type eggs.

**Reference level**	**Term**	**Estimate**	**SE**	***p*-Value**	**Odds ratio**	**95% CI**
	*Anoplocephala-*serum positive	0.483	0.338	0.15362	1.620	0.853–3.240
Always pasture access	Sometimes pasture access	−0.762	0.291	0.00884	0.467	0.258–0.812
	No pasture access	−1.950	0.501	9.87e−05	0.142	0.052–0.374
Fenbendazole	Pyrantel	−1.089	0.663	0.10056	0.337	0.075–1.095
	Moxidectin	−5.104	1.225	3.08e−05	0.006	0.0003–0.047
	Ivermectin-Praziquantel	−1.179	0.790	0.13568	0.307	0.057–1.355
	Ivermectin	−1.366	0.660	0.03858	0.255	0.057–0.823
	Doramectin	−1.716	1.361	0.20725	0.180	0.014–4.577
	Time since treatment [weeks]	0.045	0.016	0.00482	1.046	1.016–1.082
	Age [years])	−0.040	0.015	0.00773	0.961	0.933–0.989
	(Intercept)	2.446	0.761	0.00131	11.543	2.874–60.738

*pseudo R${}_{\text{McFadden}}^{2}$: 0.16.*

*pseudo R${}_{\text{Nagelkerke}}^{2}$: 0.27*.

### Risk Factor Analysis for Seropositivity for *Strongylus vulgaris* Recombinant SvSXP Larval Antigen

**Figure 7:**
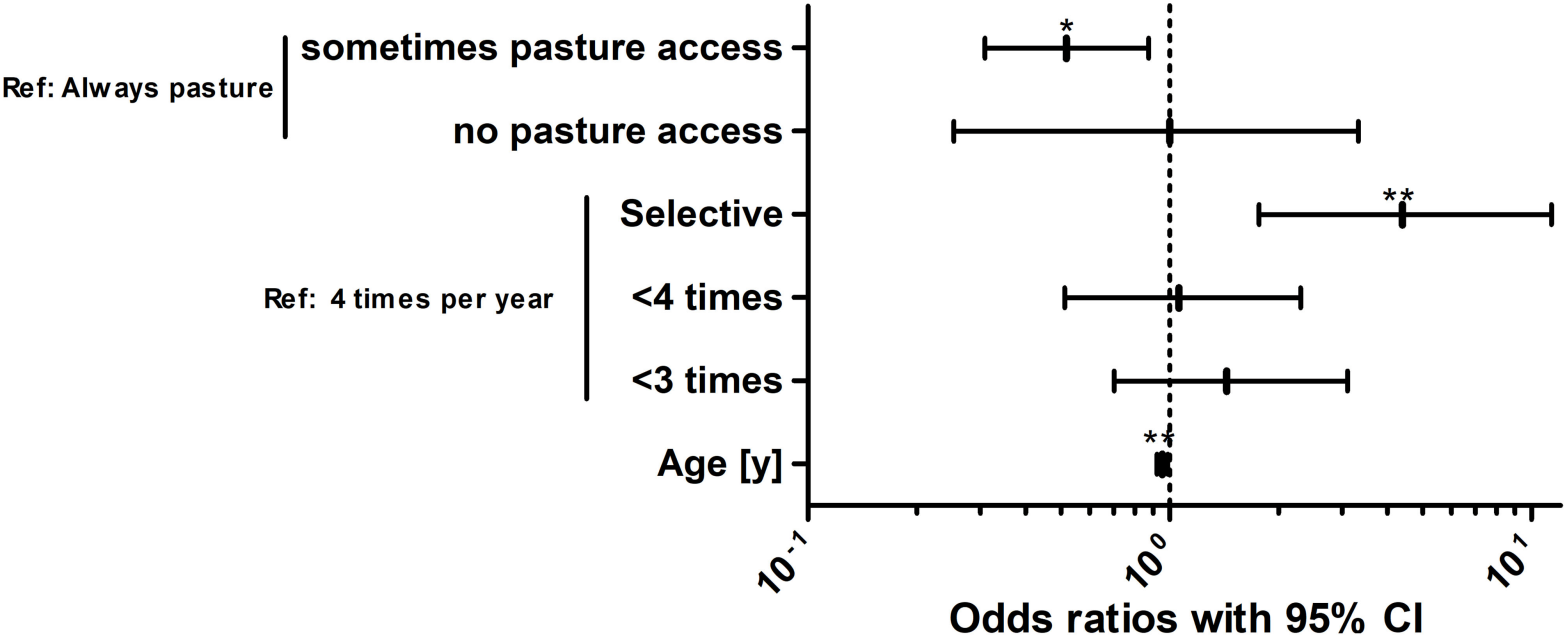
Risk factor analysis for the odds of horses in the study population to be positive for antibodies against the *Strongylus vulgaris* recombinant SvSXP larval antigen. Risk factors for the odds to be seropositive or negative for antibodies against *S. vulgaris* using logistic regression analysis. The treatment scheme refers to whether horses were selectively dewormed based on previous diagnosis (selective) or regularly dewormed 1–2.5 times (<3 times), 3–3.5 times (<4 times), or 4 times per year on average. The age of the horses was rounded into whole years [y]. For foals and yearlings, the age was converted from months to years by dividing the month measurement by the conversion ratio 12. **, *p* < 0.01; *, *p* < 0.05.

For identification of risk factors for *S. vulgaris* seropositivity, the same variables were initially considered as for the analysis of the risk to shed strongyle eggs. Animals were considered to be positive for this analysis based on the more conservative 30% ODR cut-off. After backward elimination, the optimised model included only the variables “pasture access”, “annual treatment schedule” and “age” (Figure 7, Table 6).

**Table 6:** Final logistic regression model to identify risk factors to be positive for antibodies against the *Strongylus vulgaris* recombinant SvSXP larval antigen.

**Reference level**	**Term**	**Estimate**	**SE**	***p*-Value**	**Odds ratio**	**95% CI**
Always pasture access	Sometimes pasture access	−0.658	0.265	−2.483	0.518	0.308–0.874
	No pasture access	0.0006	0.642	0.999	1.001	0.253–3.316
4 treatments per year	< 4 times	0.057	0.380	0.881	1.059	0.512–2.297
	< 3 times	0.363	0.376	0.334	1.438	0.702–3.102
	Selective	1.480	0.472	0.002	4.395	1.764–11.327
	Age [years])	−0.048	0.018	0.006	0.953	0.920–0.986
	(Intercept)	−0.595	0.476	0.211	0.551	0.212–1.381

*pseudo R${}_{\text{McFadden}}^{2}$:0.07.*

*pseudo R${}_{\text{Nagelkerke}}^{2}$:0.11*.

Regarding pasture access, limited access to pasture was protective compared to the reference of unlimited access to the pasture. Surprisingly, horses without pasture access were not significantly protected but this can be explained by the small number of only 16 horses (3.3%) in this group leading to wide 95% CIs. The odds to be positive for antibodies against *S. vulgaris* decreased gradually with increasing numbers of average annual anthelmintic treatments. However, only the difference between four annual treatments and a selective treatment scheme was significant with horses under selective treatment having an about 4.4-fold higher odds than horses that are treated four times per year. Finally, older horses had a lower risk to be positive for *S. vulgaris* specific antibodies with the odds to be positive decreasing by 0.95-fold per life year.

## Discussion

In the present study, prevalence of strongyle eggs on the individual horse as well as on the farm level were within the range observed in other studies from Europe before. High individual horse prevalences ranging up to 100% have been reported (13, 95, 96), although two studies conducted in Germany found lower prevalence on individual level: prevalences of 44.6% (15) and 55.3% (14) were determined. Concerning the true strongyle infection prevalence, the individual horse coproscopic prevalences, herein 66.7%, must always be considered against the background that only patent infections can be detected using coproscopy. Moreover, some patent infections will most likely remain undetected due to variation in egg shedding between droppings (97). More importantly, encysted larvae have been shown to represent the major part of the total cyathostomin burdens (95).

The results of the *S. vulgaris* real-time PCR, in which 0.83% of the samples were positive, are consistent with findings of previous studies that also reported a prevalence of <2% (horse level) using larval culture (14, 15) and PCR (14). The PCR HRM assay to detect *S. edentatus* and *S. equinus* identified the presence of *S. edentatus* in 10 horse samples, whereas no amplification of *S. asini* and *S. equinus* was observed. This is in line with the reported prevalences of large strongyles in other publications, since *S. vulgaris* and *S. edentatus* were reported to occur more frequently in horses than *S. equinus* (21, 98). Individual horse prevalences of 0–44% for *S. edentatus* and 0–10.9% for *S. equinus* have been reported in previous studies conducted in Germany (19–22). For technical and convenience reasons, primers were designed in a way to be also able to also amplify *S. asini*, since the complete group of *Strongylus* species would be of relevance in possible subsequent studies. Infection of horses with *S. asini* has to the knowledge of the authors not been reported from naturally infected horses and the only reported experimental infection of a single horse led to lower pathology caused by migrating larvae than in a zebra and a donkey infected in parallel (39). Clear evidence whether *S. asini* can lead to patent infection in horses is missing.

Table 4 provides details of the FEC of the horses that were tested *S. edentatus*- and/or *S. vulgaris*-positive with real-time PCRs. It is noticeable that all positive horse samples were collected between May and July 2017. No horse was tested positive for large strongyles that had been sampled in the later period (between August 2017 and January 2018). This can be explained by the life cycle of *S. vulgaris* with larvae undergoing migration over winter and sexually mature adults start shedding eggs in spring (99, 100). Later in the year, the practise of regular anthelmintic treatment during the grazing period could eliminate the adult *S. vulgaris* leading to absence of patent infections despite presence of the parasite on the farm. The majority of the study population received anthelmintic treatment at regular intervals (Table 2). For the four samples that were tested positive for *S. vulgaris*, no polymorphisms associated with BZ resistance detected in other strongyle nematodes were found herein neither using pyro- nor Sanger sequencing in the *S. vulgaris*-specific amplicons. The newly developed pyrosequencing assays can be potentially used in future epidemiological surveys to determine whether BZ resistance associated genetic changes will evolve in large strongyles similarly as it has been documented in several ruminant helminth species.

Since FECs are in most cases dominated by eggs of cyathostomins and cannot be discriminated from eggs of large strongyles in a FECRT, BZ resistance of large strongyles would not be recognised against the background of widespread BZ resistance in cyathostomins. Molecular approaches to detect large strongyles in post-treatment samples such as the real-time PCRs used here and assays to directly quantify molecular AR markers such as the novel pyrosequencing assay for *S. vulgaris* promise to help to perform AR monitoring for these highly pathogenic equine parasites.

The number of horses that were tested *S. vulgaris* positive in the ELISA was much higher than with the PCR. According to Andersen, et al. (54), there is a statistical correlation between a higher anti-rSvSXP titre and the number of migrating larvae in the mesenteric arteries. The rSvSXP-ELISA was introduced as an improved method to detect early stages of *S. vulgaris* during prepatency of an infection. The horses tested positive for *S. vulgaris* by real-time PCR had at least an ODR of 16.9% in the ELISA. Thus, all of these horses had ODRs above the original cut-off ODR of 13.5% with a sensitivity of 0.66 and a specificity of 0.82 (54).

The cut-off values for the rSvSXP-ELISA applied in the present study were 25 and 30%, which is a more conservative approach decreasing the number of false positives but also increasing the number of false negatives. High *S. vulgaris* seroprevalences were also reported in other studies (91, 101), but are unexpected in light of surveys employing direct pathogen detection methods (14, 15). The high number of serologically positive horses compared to the four horses tested *S. vulgaris* positive by real-time PCR may partly be due to the fact that early stages in the prepatency of the infection can be detected only with the ELISA. Moreover, antibody levels only decrease to background after 5 months post infection (102). Therefore, seropositivity is probably due to frequent exposure with infective larvae that start migration while patency only develops in a few horses since the developmental cycle is disrupted by anthelmintic treatment in most of the exposed animals. However, the small number of horses in which adult *S. vulgaris* develop and produce eggs is obviously sufficient to contaminate the pasture leading to high seroprevalence in the respective horse population. Another important issue that needs to be taken into consideration is a potential cross-reactivity of rSvSXP with the orthologous protein from other *Strongylus* species such as *S. edentatus* and the cyathostomins, which appear to be phylogenetically closely related but have not been characterised for most cyathostomin species (54).

The multivariate risk factor analysis (logistic regression) revealed that the odds to shed strongyle nematode eggs were significantly increased with a longer period between the last anthelmintic treatment and sampling. This is expectable since chances to be reinfected increase over time. In addition, the analysis indicated that the odds of shedding strongyle type eggs increases with an *Anoplocephala* spp. positive serological ELISA but this effect was not significant. This variable probably does not directly influence the odds and represents a confounder since both parasite groups share transmission via grazing. Therefore, it is also reasonable that limited/no access to pasture was protective, because it means lower exposure to infectious larval stages. Age as protective factor is consistent with the perception of young horses showing a higher incidence of patent strongyle infections due to age-dependent immunity developing through continuous exposure (103, 104). With ivermectin and particularly strong with moxidectin, the anthelmintic used for the last treatment had a protective effect compared to the reference fenbendazole. Treatments with ivermectin plus praziquantel, pyrantel, and doramectin treatments were also protective but the effects were not significant. For ivermectin and ivermectin plus praziquantel the odds ratios were similar but the effect for the latter was not significant, possibly since only a very small number of horses was treated with the combination. Doramectin was used only for a few horses from a single farm and it must be clearly stated that doramectin is not licenced as a dewormer of horses and that the ruminant product was used. Resistance to BZs and pyrantel is well-known to occur in many European countries (61, 63, 105) including Germany (64) at high prevalence. This might lead to treatment failures (faecal egg count reduction below 90–95%) (57) or to shorter egg reappearance periods (106). Resistance to ivermectin and particularly moxidectin in cyathostomins have only rarely been reported on a global scale but so far not in Germany.

The logistic regression analysis of the data on *S. vulgaris* seroprevalence identified only a few variables that had significant effects on the odds to be positive, i.e., “pasture access”, “treatment scheme” and the age of the horses. Regarding “pasture access”, only “sometimes pasture access” was significantly protective in comparison to “always pasture access” while “no pasture access” was surprisingly not protective. The odds ratio was close to 1 but the 95% CI was also very wide which is due to the fact that only 16 horses (3.3%) were assigned to this category. Increasing age was again slightly protective, which can be explained by immunity slowly developing over time. In contrast to the analysis on the odds to be positive for shedding strongyle eggs, the treatment scheme had a significant effect on the odds to be positive for antibodies against the *S. vulgaris* larval antigen. There was a clear tendency that higher treatment frequencies were associated with lower odds to have antibodies against rSvSXP. However, only the difference between four treatments per year and selective treatment was significant. Using morphological analysis of L3 from larval cultures, Nielsen et al. (84) have previously reported that selective deworming strategies resulted in roughly doubled prevalences of *S. vulgaris* on the individual animal and farm level in Denmark. It is also remarkable that in contrast to the analysis of data on positivity of faecal samples, the drug used for the last treatment was excluded from the final regression model for seropositivity for the larval *S. vulgaris* antigen since it led to an increased AIC. This substantiates the idea that the odds to be positive for antibodies against *S. vulgaris* are more dependent on the contamination of the pasture (and thus the treatment history of the farm) and not on the treatment history of the individual horse since for immune reactions and seropositivity the exposure is relevant and not the risk to develop a patent infection. Out of the 42 horses for which the owners reported “selective treatment”, 41 were from only 4 farms on which all horses were assigned to the “selective treatment” group. This confirms that the “selective treatment” scheme was highly clustered on the farm level.

## Conclusion

PCR assays identified *S. vulgaris* and *S. edentatus* at only low frequencies, which is in contrast to the high *S. vulgaris* seroprevalence observed herein. The cause for the very high prevalence of antibodies against rSvSXP remains unclear and should be further examined to exclude any confounders such as cross reactivity. The fact that an important risk factor for being positive for antibodies against a larval *S. vulgaris* antigen was a selective treatment scheme based on prior diagnosis, emphasises the risk of such treatment schemes, if they do not include regular analysis of eggs or larvae or antibodies for the presence of *Strongylus* spp. on a farm.

## Data Availability Statement

The raw data supporting the conclusions of this article will be made available by the authors, without undue reservation.

## Ethics Statement

The animal study was reviewed and approved by Landesamt für Gesundheit und Soziales, Berlin, registration no: Reg 0059/17. Written informed consent was obtained from the owners for the participation of their animals in this study.

## Author Contributions

LJ, JK, EB, JB, MN, and GS-H: study concept and design. LJ: sample collection and Mini-FLOTAC and PCR analyses. NH: involved in establishment of the *Strongylus* spp. HRM PCR. GS-H funding acquisition and project management. LJ and JK: data analyses and drafting the first version of the manuscript. All authors contributed to the article and approved the final manuscript version.

## Funding

This work was funded by Virbac in a research collaboration with Freie Universität Berlin (contract number 2014000271).

## Conflict of Interest

JB is employed by Virbac Tierarzneimittel GMBH and EB by Virbac France. GS-H declares that he has repeatedly acted as consultant for veterinary pharmaceutical and diagnostic companies and has previous and ongoing research collaborations with various companies. The remaining authors declare that the research was conducted in the absence of any commercial or financial relationships that could be construed as a potential conflict of interest.

## Publisher's Note

All claims expressed in this article are solely those of the authors and do not necessarily represent those of their affiliated organizations, or those of the publisher, the editors and the reviewers. Any product that may be evaluated in this article, or claim that may be made by its manufacturer, is not guaranteed or endorsed by the publisher.
