# Supplementary material for: Occurrence of Strongylid Nematode Parasites on Horse Farms in Berlin and Brandenburg, Germany, With High Seroprevalence of Strongylus vulgaris Infection
Source: Front Vet Sci. 2022 Jun 10;9:892920. doi: 10.3389/fvets.2022.892920 (PMC9226773; doi:10.3389/fvets.2022.892920)
Supplement: Supplementary file 1 [file Data_Sheet_1.PDF]

Supplementary Material

Article

Occurrence of strongylid nematode parasites on horse farms in Berlin and Brandenburg, Germany with high seroprevalence of *Strongylus vulgaris*

Questionaire S1. Questionaire to obtain information regarding treatment history and management on the horse farms.

Ⅰ. Horse keeping

1. Farm type:

   ☐ Riding stable (less than 2 foals, more than 15 horses)

   ☐ Small farm (less than 2 foals, less than 15 horses)

   ☐ Stud farm (more than 2 foals)

2. Total number of horses:........................

3. Age of horses: <u>Quantity</u>

   Foals (under 1 year):...............

   Yearlings (1-3 years):............

   Adults (over 3 years):............

4. Horse accomodation:

   ☐ Open stable

   ☐ Box

   ☐ Group Box

   ☐ other

Ⅱ. Pasture management

1. Do the horses have access to the pasture? ☐yes ☐no

   How often?

   ☐ year around

☐ only in summer

☐ full day

☐ a few hours

2. How many hectares of pasture are used per horse?..............................
3. Are pastures regularly changed? ☐ yes ☐no

   If yes, at what intervals?...

   After anthelmintic treatment? ☐ yes ☐no
4. Do you feed fresh (chopped) grass? ☐ yes ☐no

Ⅲ. Hygiene – Stable and pasture

1. How often do you clean the stables?..
2. Do you disinfect your stable annually? ☐ yes ☐no
3. If yes, what disinfectant is used?..
4. Is cleaning or disinfection carried out after deworming?

   ☐ yes ☐no
5. Do you collect faeces from the pastures? ☐ yes ☐no

   How often?...
6. Do you use hay racks? ☐yes ☐no

Ⅳ. Anthelmintic management

1. Are horses dewormed at regular intervals? ☐yes ☐no

   Do all horses receive anthelmintic treatment at the same time? ☐yes ☐no

   Date of the last anthelmintic treatment:..

   Which drug was used?

   foals: ....

   yearlings:...

   adults:...

   breeding mares:...
2. How often is anthelmintic treatment given?:..

3. Do you change the drug classes regularly?

    ☐yes ☐no

    How often?...

4. Are new horses dewormed before arrival at the farm? ☐yes ☐no
5. Do you follow an evidence-based selective deworming approach based on faecal egg counting?

    ☐yes ☐no

    Which faecal egg count is the limit above which the horses are treated?

6. Do you monitor the success of the anthelmintic treatment using faecal samples?

    ☐yes ☐no

7. Please describe the anthelmintic management: ....

    ....

Many thanks

Jürgenschellert, L., Krücken, J., Austin, C.J., Lightbody, K.L., Bousquet, E., von Samson-Himmelstjerna, G., 2020. Investigations on the occurrence of tapeworm infections in German horse populations with comparison of different antibody detection methods based on saliva and serum samples. Parasites & Vectors 13, 1-10.

Supplementary Table 1 | Primers used for diagnosis of *Strongylus* spp.

Sequence (5' - 3')	Name	Target species
TATACATTAAATAGTGTCCCCCATTCTAG	*S_vulgaris*_for (modified based on Nielsen (2008))	*Strongylus vulgaris*
GCAAATATCATTAGATTTGATTCTTCCG	*S_vulgaris*_rev (Nielsen (2008))	*Strongylus vulgaris*
AATCCCCATTCTAGAAAAGAAT	S.eden:equi.asin.HRM.for	*Strongylus edentatus*, *Strongylus equinus*, *Strongylus asni*
ATAAGTCGGTAAACAATTTAGC	S.eden:equi.asin.HRM.for	*Strongylus edentatus*, *Strongylus equinus*, *Strongylus asni*

Supplementary Table 2 | Primers for pyrosequencing assays for *Strongylus vulgaris* isotype 1 β-tubulin gene

Sequence (5‘ to 3‘)	Name
CGGGTATGGGAACTCTCCTTA	Sv_betaT_Iso1_167_F1
CGTGTTCCATTCCCAAGAACT	Sv_betaT_Iso1_167_R1
Biotin-CGTGTTCCATTCCCAAGAACT	Sv_bT_Iso1_167_P_R1
TGATAGAATCATGTCTTCGT	SvbTIso1_167S
TTTTTCTAGGTTTCCGACACTGT	Sv_betaT_Iso1_198_200_F1
AAGGTACGGAAGCAAATATCATAC	Sv_betaT_Iso1_198_200_R1
Biotin-AAGGTACGGAAGCAAATATCATAC	Sv_bT_Iso1_198_200_P_R1
GCTAGTTGAAAATACAGATG	SvbTIso1_198_200S
TTGATTGCTAAAATCCGAGAAGAG	Sv_betaT_Iso2_167_F1
CATAGAGTCCAAGCGTGCATTA	Sv_betaT_Iso2_167_R1
CCCTTTGTAATCGGATATTGCA	Sv_betaT_Iso2_198_200_F1
AAAGTTCGGAAGCAGATGTCGTA	Sv_betaT_Iso2_198_200_R1
